# Calcium Sensors STIM1 and STIM2 Regulate Different Calcium Functions in Cultured Hippocampal Neurons

**DOI:** 10.3389/fnsyn.2020.573714

**Published:** 2021-01-05

**Authors:** Liliya Kushnireva, Eduard Korkotian, Menahem Segal

**Affiliations:** ^1^Department of Neurobiology, The Weizmann Institute, Rehovot, Israel; ^2^Faculty of Biology, Perm State University, Perm, Russia

**Keywords:** dendritic spines, filopodia, store operated channels, STIM, hippocampal culture, cytosolic calcium, calcium stores

## Abstract

There are growing indications for the involvement of calcium stores in the plastic properties of neurons and particularly in dendritic spines of central neurons. The store-operated calcium entry (SOCE) channels are assumed to be activated by the calcium sensor stromal interaction molecule (STIM)which leads to activation of its associated Orai channel. There are two STIM species, and the differential role of the two in SOCE is not entirely clear. In the present study, we were able to distinguish between transfected STIM1, which is more mobile primarily in young neurons, and STIM2 which is less mobile and more prominent in older neurons in culture. STIM1 mobility is associated with spontaneous calcium sparks, local transient rise in cytosolic [Ca^2+^]_i_, and in the formation and elongation of dendritic filopodia/spines. In contrast, STIM2 is associated with older neurons, where it is mobile and moves into dendritic spines primarily when cytosolic [Ca^2+^]_i_ levels are reduced, apparently to activate resident Orai channels. These results highlight a role for STIM1 in the regulation of [Ca^2+^]_i_ fluctuations associated with the *formation* of dendritic spines or filopodia in the developing neuron, whereas STIM2 is associated with the *maintenance* of calcium entry into stores in the adult neuron.

## Introduction

Calcium stores assume a critical role in the handling of cytosolic calcium concentration ([Ca^2+^]_i_) in neurons and non-neuronal cells alike (Verkhratsky, 2005; Zalk et al., 2007). The main intracellular calcium-accumulating organelle is the endoplasmic reticulum (ER). Release of calcium from ER is important for cases, where the influx of calcium ions from the extracellular space is not sufficient to raise [Ca^2+^]_i_, to levels needed to activate calcium-dependent protein phosphorylation, or in cases where there are no sufficient calcium channels on the plasma membrane such as in juvenile neurons. Depletion of calcium from the ER stores is sensed by stromal interaction molecule (STIM). It clusters near the depleted store, relocates to the plasma membrane, where it interacts with Orai1 voltage-independent calcium channel, to allow calcium influx into the stores, to refill them (Bogeski et al., 2012; Segal and Korkotian, 2014; Kraft, 2015). The interaction of stores/STIM/Orai complex was studied extensively in non-neuronal cells, and its malfunction has been implicated in immunological diseases (Feske et al., 2006). Compared to the vast literature on STIM/Orai functions in non-excitable cells, much less is known about their role in central neurons. STIM and Orai are localized in brain tissue (Skibinska-Kijek et al., 2009; Segal and Korkotian, 2016), and STIM1/Orai1 can be converted from a dispersed to a punctate form upon depletion of calcium stores with thapsigargin (Klejman et al., 2009). They are important in the regulation of growth cone motility (Mitchell et al., 2012), in the regulation of voltage-gated calcium channels (Park et al., 2010), and in detrimental effects of chronic epilepsy (Steinbeck et al., 2011) and oxidative stress (Henke et al., 2013). Earlier work ascribed a role for store-operated calcium entry (SOCE) also in synaptic plasticity, in that SOCE antagonists reduce long-term potentiation in hippocampal neurons (Baba et al., 2003). The recent association of septins (Tada et al., 2007; Sharma et al., 2013) with SOCE is intriguing indeed, as septins have been found in dendritic spines of central neurons (Xie et al., 2007) and may provide a link between dendritic spines and calcium stores. Another peptide that regulates the activity of STIM1 is NEUROD2 (Guner et al., 2017) which is found in cortical neurons and attests to the importance of STIM1 in the regulation of stored calcium. In earlier studies, we explored the role of Orai1 in dendritic spine growth and plasticity (Korkotian et al., 2017; Tshuva et al., 2017) to suggest that Orai1 is critically involved in intracellular calcium regulation, specifically in dendritic spines. The present study focuses on STIMs. There are two species of STIM in central neurons, STIM1 and STIM2, and their possible differential roles in calcium stores are not entirely clear. While studies associate STIM1 with Orai1 (e.g.,Skibinska-Kijek et al., 2009; Pavez et al., 2019), there are indications that STIM2 is the dominant species in hippocampal neurons (Sun et al., 2014; Zhang et al., 2015). We were able to detect both STIM1&2 in cultured neurons (Korkotian et al., 2017), but possibly in different neuronal compartments and/or developmental stages of the neurons. In the present study, we focus on the dynamics of both STIMs concerning intracellular calcium regulation. Our results indicate that unlike STIM2, STIM1 predominates in young cells; it is highly mobile within dendrites and has a potential role in causing an influx of calcium through apparent calcium sparks (Ross, 2015) and in the formation and growth of filopodia, as suggested before for growth cones (Mitchell et al., 2012; Pavez et al., 2019). STIM2, on the other hand, is active in the more mature neurons, where it is mobilized in response to a reduction of ambient [Ca^2+^]_i_ and moves into dendritic spines.

## Materials and Methods

### Cultures

Animal handling was as per the guidelines of the Institutional Animal Care and Use Committee of the Weizmann Institute and with the Israeli National guidelines on animal care. Cultures were prepared as detailed elsewhere (Korkotian et al., 2017). Briefly, E18–19 rat embryos were removed from pregnant decapitated mother’s womb under sterile conditions. The hippocampi were dissected free and placed in a chilled (4°C), oxygenated Leibovitz L15 medium (Gibco) enriched with 30 mM glucose and gentamicin (Sigma, 20 μg/ml), and mechanically dissociated. About 10^5^ cells in 1 ml medium were plated on 13 mm circular glass coverslips in each well of a 24 well plate. Cells were left to grow in the incubator at 37°C, 5% CO_2_.

Neurons were transfected at 6–7 days *in vitro* (DIV) with EBFP2 or EGFP (to image cell morphology), and STIM1-mCherry or STIM2-YFP using lipofectamine 2000 and were used for imaging at 10–20 DIV.

### Immunostaining

**Figure 1:**
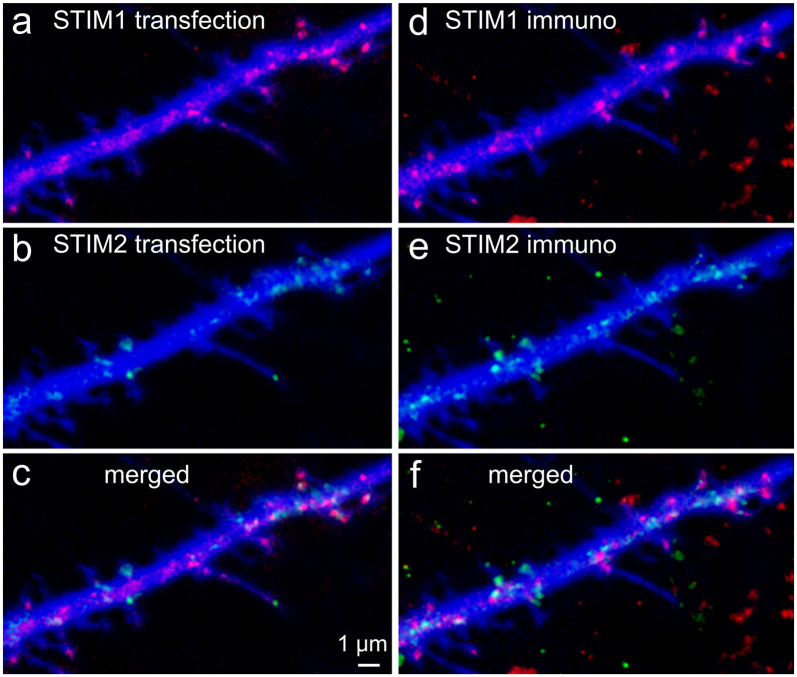
Colocalization of transfected stromal interaction molecule 1 (STIM1) or STIM2 with native STIMs identified by immunocytochemistry. Cells were transfected at 7 days *in vitro* (DIV) and fixed in PFA at 10 DIV. Secondary antibodies were Cy2 anti-rabbit and Cy5 anti-goat. Lasers and channels were distributed as follows: BFP (cell morphology marker) 405 nm (blue), Cy2 (STIM1, anti-rabbit) 458 nm, STIM2 + YFP 514 nm, STIM1 + mCherry 543 nm, Cy5 (STIM2, anti-goat) 633 nm. Overall, in the figure, STIM1 is shown in red and STIM2 in green. 3D-reconstructed Z-stacks, slow, high-resolution imaging mode, separate imaging tracks, and the GASP detector of Zeiss 880 were used for best dye/staining separation. Partial overlap of the transfected species with the immunocytochemically detected species was clear for STIM1 (**A,D**, respectively) and was different from those of STIM2 **(B,E)**. It should be noted that some transfected STIM1&2 puncta **(A)** were not detected in the immunostaining for STIM1&2 **(D)**, probably because the antibody detects fewer puncta in the fixed tissue, unlike the transfected species that is imaged in-toto. This can be seen in the merged images **(C,F)**.

Cover glasses bearing transfected primary hippocampal cells were washed briefly with a standard extracellular solution. Cultures were then fixed with 4% paraformaldehyde in 0.1 M phosphate-buffered saline (PBS, pH 7.4) for 20 min, and washed thereafter with PBS thoroughly. Cultures were incubated for 1 h with 10% normal horse serum (NHS) in 0.1% Triton X-100 containing PBS and subsequently incubated for 24 h at 4°C with the specific antibodies. Anti STIM1 (Santa Cruz, rabbit IgG, 1:200) and anti STIM2 (goat polyclonal IgG, Santa Cruz, 1:200) were combined in different testing conditions. Cultures were incubated for 1 h with Alexa 568-labeled or Alexa 633-labeled anti-goat or anti-mouse secondary antibody (Molecular Probes, Eugene, OR, USA; 1:200, in PBS). Coverslips were rinsed, transferred onto glass slides, and mounted for visualization on a Zeiss upright LSM 880 (which allows simultaneous visualization of four fluorophores) with an anti-fading mounting medium. In all cases, secondary and tertiary dendritic segments were visualized. It should be noted that although STIM1 (Figures 1A,D) and STIM2 (Figures 1B,E) share some structural homology, they were physically located in distinctly different cellular compartments, indicating that their antibodies express low cross-reactivity (Figures 1C,F). Confocal image stacks were used for 3D reconstructions using Zeiss software. Figures were prepared using Photoshop CS4 graphics software (Adobe, San Jose, CA, USA).

### Live Cell Imaging

Cultures were placed in the imaging chamber, on the stage of the confocal microscope using a 40× water immersion objective (1.0 NA) and imaged at a rate of 10–20 frames/s. No photo-bleaching was detected under these conditions. Standard recording medium contained (in mM); NaCl 129, KCl 4, MgCl_2_ 1, CaCl_2_ 2, glucose 10, HEPES 10, pH was adjusted to 7.4 with NaOH and osmolality to 320 mOsm with sucrose. Cultures were incubated with Fluo-2AM or Calcium Orange AM (CO; 2 μM, Invitrogen) for 1 h at room temperature to image variations in [Ca^2+^]_i_ resulting from spontaneous network activity or changes associated with STIM1 or STIM2. Imaging of cell morphology (blue, imaged at 405 nm), calcium variations (488 nm or 543 nm), and STIM1/2 (514/543 nm) were made with the appropriate wavelengths. Images were taken in the fast scan mode of the Zeiss 880 confocal microscope, using a two-track setting, in which first the 488 nm illumination was applied alone, and then a combination of 405 and 543 nm was used. This setting minimizes the cross-talk between different dyes and still allows the fast scan necessary for calcium imaging. Pinhole size was adjusted to about 2 μm. All measurements were conducted with identical laser parameters for all groups (e.g., intensity, optical section, duration of exposure, and spatial resolution). The immunostained cells were carefully examined and all cases of overlapping of two or more cells were discarded. Only well-identified cells and their segments were analyzed. In each randomly selected segment of 50 μm in length, all present protrusions were identified and counted (without preselection). They were expected to be fewer in the younger segments than in the older ones. The analysis was carried out in optical sections with a thickness of 0.8 μm. The total number of fragments with protrusions was taken as 100%. Then the similar short areas without any processes were distinguished. We tried to keep their number equal to the number of fragments with processes. This was also taken as 100%. After that, each of the dendritic fragments (with or without a process), was carefully examined for the presence of STIM1 or STIM2 puncta. The number of positive cases was expressed as a percentage of the total. The reliability of the assay was ensured by randomly selected cells/segments, careful optical sectioning, and high-quality immunostaining.

### Statistical Analysis

Fluorescent intensity was measured using ImageJ (NIH, USA) and the MATLAB (R2010b, USA)-based line-scan acquisition program. Measurements were made in a double-blind procedure (in some cases even with three independent observers) to assure unbiased observations. Dendritic protrusions were categorized either as spines, consisting of a clear head, usually larger than 0.5 μm in diameter and a short neck, or filopodia, long protrusions which are devoid of a discernable head. Dendritic spines that were used for calcium imaging were identified in the BFP-transfected neurons and analyzed independently of the measurements of calcium transients in these same spines. Statistical comparisons were made using *t*-tests or ANOVA, as the case requires, using MATLAB and KaleidaGraph software.

## Results

**Figure 2:**
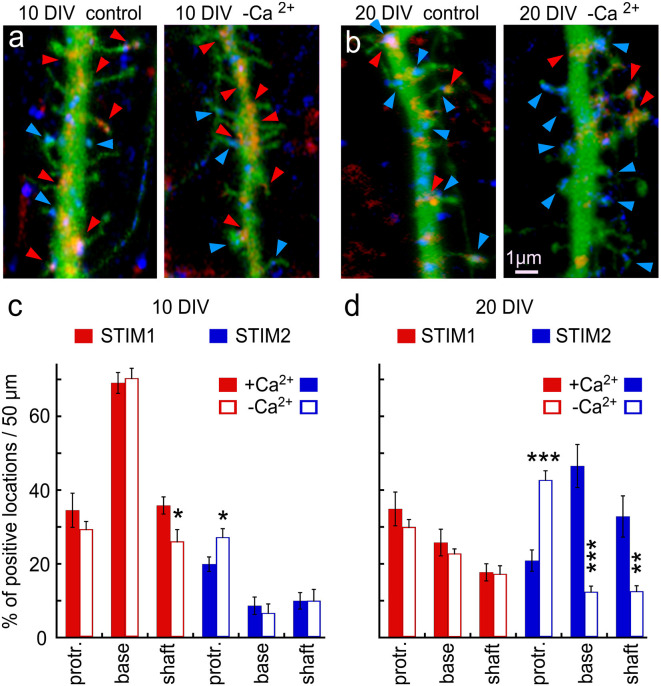
Immunohistochemical localization of STIM1&2 in cultured hippocampal neurons with and without extracellular calcium. **(A,B)** Sample dendrites were taken from 10- to 20-day-old cultures and stained for STIM1(red) and STIM2 (blue) in cells transfected with EGFP (green) to visualize morphology in the presence and absence of extracellular calcium. It is apparent that the 10-day-old culture contains more STIM1 than STIM2 puncta, and the opposite is seen in the 20-day-old neuron. Under the calcium-free condition, STIM2 flows into protrusions in young and, especially, old culture. **(C)** Bar graphs quantification of the results illustrated on the left. The difference between STIM1 and 2 in 10 DIV in both conditions is highly significant (control conditions: *n* = 10 dendrites from five cells for each group, ANOVA *p* < 0.0001; in calcium-free medium: *n* = 10 dendrites from five cells for each group, ANOVA *p* < 0.0001). **(D)** Bar graphs quantification of the results illustrated on the right. The difference between STIM1 and 2 in 20 DIV in both conditions is highly significant (control conditions: *n* = 10 dendrites from five cells for each group, ANOVA *p* < 0.0002; in calcium-free medium: *n* = 10 dendrites from five cells for each group, ANOVA *p* < 0.0001). *Significant, 0.05 > *p* > 0.01; **very significant, 0.01 > *p* > 0.001; ***highly significant, *p* < 0.001.

Since others and we have identified both STIM1 and STIM2 in hippocampal neurons, we explored the possibility that they are located at different neuronal compartments or possibly that they are expressed at different developmental ages and geared for different functions. Indeed, there was a striking age-dependent difference in presence of STIM1&2 in dendritic protrusions (filopodia and spines), such that STIM1 was more prevalent in the 10 days *in vitro* cells (10 DIV), compared to STIM2 (Figure 2), and the other way around for the 20 DIV neurons. This indicates that STIM1 may have a role in neuronal development and synapse formation, unlike STIM2, which may function in the maintenance and regulation of store-operated currents in the adult neurons.

**Figure 3:**
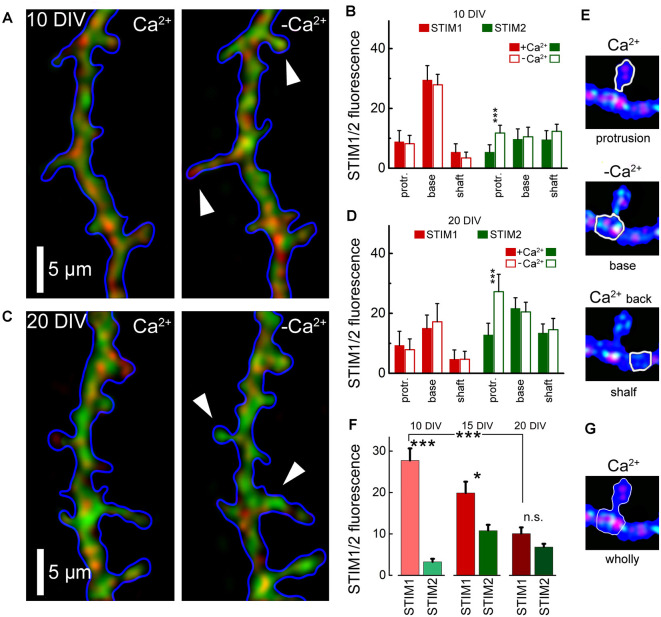
Averaged STIM 1&2 fluorescence in protrusion, base, and shaft dendrites, in normal medium (2 mM Ca) and 15 min after incubation with a calcium-free medium. **(A)** STIM1 (red) and 2 (green) fluorescence in cell incubated with and without calcium, 10 DIV. **(B)** Bar graphs: averaged fluorescence minus background for in each group, 10 DIV. The difference between STIM1 and 2 in the base in both conditions and the difference between STIM2 with and without calcium in protrusion is highly significant (*n* = 10 dendrites from six cells for each group, ANOVA *p* < 0.001). **(C)** STIM1 (red) and 2 (green) fluorescence in a medium with and without calcium, 20 DIV. **(D)** Bar graphs: averaged fluorescence minus background for in each group, 20 DIV. The difference between STIM1 and 2 in the base in both conditions is not significant, but the difference between STIM2 with and without calcium in protrusion is highly significant (*n* = 8 dendrites from four cells for each group, ANOVA *p* < 0.001). **(E)** An example of the moving of STIM1/2 puncta in a protrusion (spine) in medium with and without calcium and after the return of calcium back to normal. White arrows mark protrusions in which the influx of STIM2 in a calcium-free medium is most noticeable (**A,C**, right panels). **(F)** STIM1 and STIM2 fluorescence at the base with protrusions (filopodia or spines) with background subtracted, at 10 DIV, 15, and 20 DIV. DIV 10: 34 protrusions; DIV 15: 30; DIV 20: 35 protrusions. **(G)** An example fluorescence calculation of STIM1/2 puncta at the base with protrusions (spine) for **(F)**. *Significant, 0.05 > *p* > 0.01; ***highly significant, *p* < 0.001; n.s., not significant, *p* ≥ 0.05.

To further explore the role of STIM1 in the formation of dendritic protrusions, we time-lapse imaged at high-resolution dendrites of cultured neurons at 10 and 20 DIV (Figure 2). At a younger age, the dendrites are motile and new protrusions, spines, and filopodia emerge and disappear. Comparing two time-points, with and without Ca^2+^ (after 15 min) of the same dendrites, we found that new filopodia at 10 DIV, are endowed with STIM1 puncta (Figures 2A,C). This was not the case for dendritic spines, which are much less motile compared with filopodia, and are less associated with STIM1 in the more mature neurons (Figures 2B,D). The age-dependent difference between STIM1 and 2 was seen clearly in the live tissue, not subject to fixation (Figure 3), clearly indicating that this disparity is genuine and that only STIM2 responds to a reduction in ambient [Ca^2+^]_o_.

**Figure 4:**
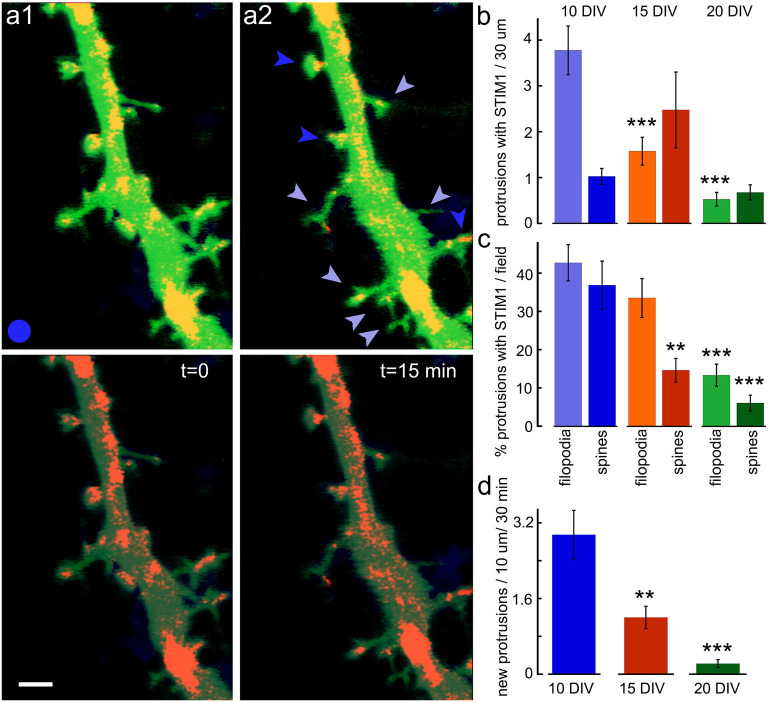
New protrusions are associated with STIM1. **(A1)** Puncta are shown at two time-points; *t* = 0 and **(A2)** *t* = 15 min. Top images are EGFP (green, for morphology) and STIM1-mCherry (red) merged. Bottom panels show the same fields with EGFP dimmed. Scale bar = 2 μm. **(B)** The overall ratio of STIM1-containing protrusions in 10, 15, and 20 DIV is shown in absolute values. The total number of protrusions per standard field (filopodia and spine-like structures) are shown for all three ages (*n* = 40 fields for each age). **(C)** The normalized proportion of STIM1-positive protrusions as a percent of the total number of protrusions per field. **(D)** The appearance of new protrusions, associated with STIM1 puncta for DIV 10, 15, and 20 in the standard field per 30 min is summarized. **Very significant, 0.01 > *p* > 0.001; ***highly significant, *p* < 0.001.

The association of STIM1 with the morphological changes in the host neuron was studied using time-lapse imaging of dendritic segments in the presence of STIM1 puncta (Figure 4). The young neurons are far more dynamic than the older ones, and new filopodia and spines can be formed and eliminated within 15 min of imaging. In the intermediate aged neurons (15 DIV) both filopodia and novel spines can be formed within 15 min of observations, and far fewer protrusions are seen in the 20 DIV cells. In all cases, the majority of novel protrusions are associated with the presence of STIM1 puncta.

**Figure 5:**
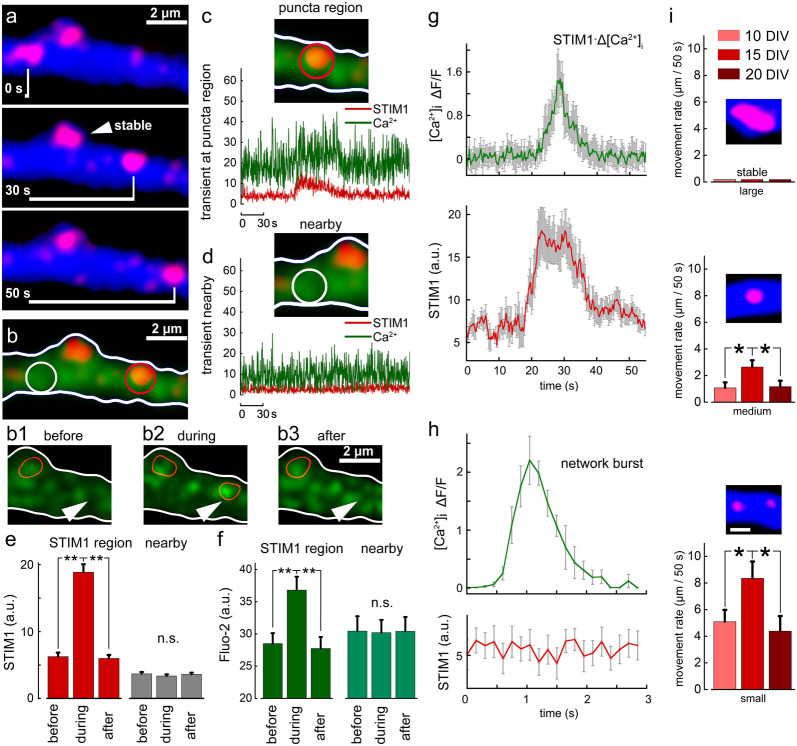
Motility of STIM1 puncta along a dendrite and their association with a transient rise of [Ca^2+^]_i_. **(A)** Three images of a dendritic segment containing one stable STIM1 puncta, and a mobile one moving from left to right. **(B)** Fluo2 sensor shows calcium transients. Panels **(B1–3)** show calcium transient in the area, marked with an arrowhead. The current location of the mobile STIM1 punctum is marked with a red contour. The second red contour corresponds to the stable STIM1 punctum. **(C)** When punctum is present at the red location on the right a calcium transient can be detected. **(D)** In an adjacent location, neither puncta nor calcium transient is seen. Averaged data, *n* = 10 regions from different cells and different cultures. Three time points are taken: right before the entrance of puncta into the selected region (before) in the presence of puncta within the region of interest (during) and right after the puncta left the region (after). Simultaneously, measurements from a randomly selected nearby region were made (nearby). STIM1 for these time points (**E**, red). Nearby region (**E**, gray). Calcium for these time points (**F**, dark green). Calcium measurements from a nearby region (**F**, light green). **(G)** Time course of change in [Ca^2+^]_i_ fluorescence at the STIM1 puncta (top, green) and the STIM1 fluorescence (red, low trace) of 10 dendrites. Note that STIM1 fluorescence precedes the rise of [Ca^2+^]_i_ and is sustained after the calcium change subsided. **(H)** A fast synapse-evoked [Ca^2+^]_i_ rise that is not associated with a change in STIM fluorescence. Note the much faster time course of change in the synaptic event compared to the change seen above. **(I)** Movement rates (μm/50 s of three sizes of STIM1 puncta (large: >2 μm, medium: 1–2 μm and small: <1 μm), at three age groups, 10, 15 and 20 DIV. Small puncta move faster than medium and large ones at all age groups (*p* < 0.05). The faster movement rate is observed at 15 DIV compared to 10 and 20 DIVs for both medium and small-sized STIM1 puncta (medium: *n* = 27, 19 and 17 for 10, 15 and 20 DIV, respectively, *F* = 3.58, *p* < 0.05; small: *n* = 27, 14 and 8, *F* = 3.02, *p* < 0.05). *Significant, 0.05 > *p* > 0.01; **very significant, 0.01 > *p* > 0.001; n.s., not significant, *p* ≥ 0.05.

In earlier experiments, we found that STIM1 puncta are mobile along the dendrites, and occasionally enter dendritic spines and filopodia. We now explored the possible association of STIM1 mobility with local fluctuation of ambient [Ca^2+^]_i_. To examine this, we followed STIM1 puncta using time-lapse photography to find that they move along dendrites at a slow rate (Figure 5A). Simultaneous measurement of ambient [Ca^2+^]_i_ clearly indicates that passage of STIM1 puncta in the field of view is associated with a transient local rise of [Ca^2+^]_i_. This local rise follows by 5–10 s the entry of STIM1 puncta into the field of view (Figures 5B1–3). Such local rise of [Ca^2+^]_i_ is different from that produced by a large synchronized, back-propagating action-potential or a transient local synaptic spike (Figures 5G,H) which has a fast rise time, less than 0.5 s to peak and fast decay, but is not related to STIM1 motility. This change is local and is not seen in regions adjacent to the traveling STIM puncta (Figures 5C–F). The smaller the size of the STIM1 puncta, the more motile it is (Figure 5I).

**Figure 6:**
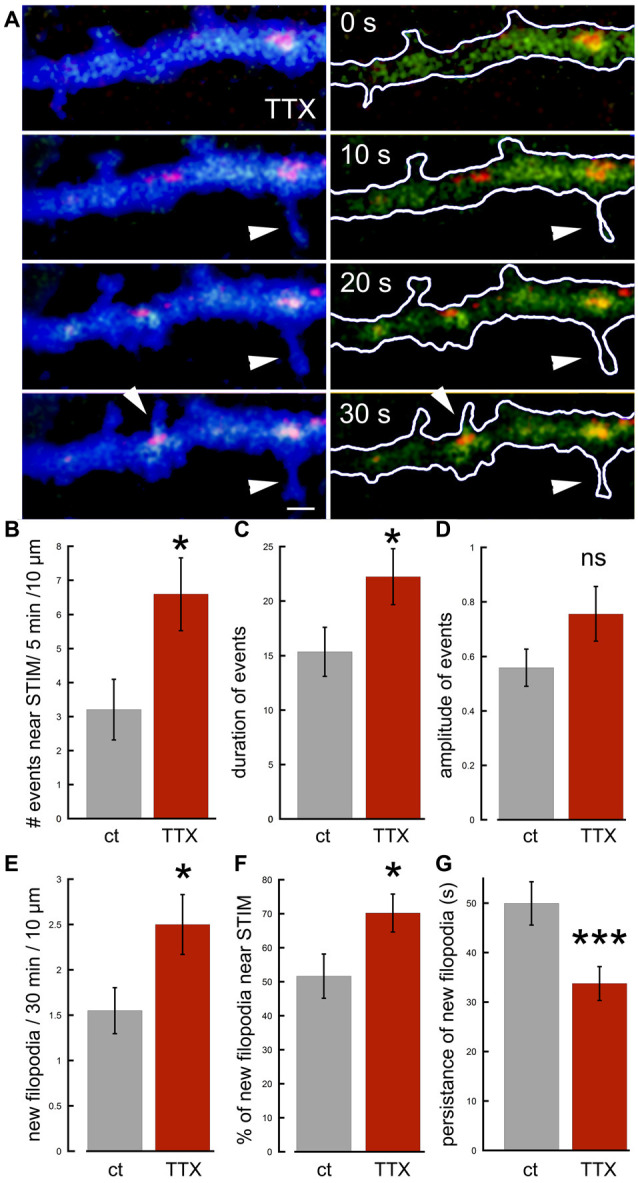
Effects of tetrodotoxin (TTX) on STIM1-associated calcium transients and the formation of nascent filopodia. **(A)** Sample illustration of a dendrite at four time-points, 10 s apart, from top to bottom, showing calcium transients (green), associated with STIM1 puncta (red) in EBFP-transfected neuron (blue), recorded in TTX. Some calcium transients in the dendrite are associated with filopodia outgrowth (arrowheads). **(B)** The number of calcium transients during 5 min per standard segment is measured in control and TTX (*n* = 20 fields, seven cells for each). **(C)** Averaged duration of events (*n* = 40 events, 20 fields for each) scale = secs. **(D)** Averaged amplitude of events (*n* = 40 events, 20 fields for each). **(E)** All related to filopodia outgrowth: new filopodia per 30 min per standard segment, associated with STIM1 puncta and calcium transients (*n* = 40 fields, seven cells for each). **(F)** Percent of new filopodia associated with STIM1, of total new filopodia per standard field (*n* = 40 fields). **(G)** Averaged persistence of new filopodia in time (seconds; *n* = 55 protrusions for each). *Significant, 0.05 > *p* > 0.01; ***highly significant, *p* < 0.001; n.s., not significant, *p* ≥ 0.05.

To clarify the distinction between local and generalize calcium transients even further, and to examine the role of synaptic activity and network-related formation of dendritic spines and filopodia, we compared STIM-associated calcium transients in control and tetrodotoxin (TTX)-treated cultures, where network activity is aborted. Surprisingly, there were a significantly larger number of STIM1-associated calcium transients, as well as the formation of short-lived filopodia in presence of TTX compared to control (Figure 6). The morphological results, showing that TTX triggers elongation of spines are similar to previous observations on the acute effects of TTX on the formation of spinules (Verbich et al., 2016).

**Figure 7:**
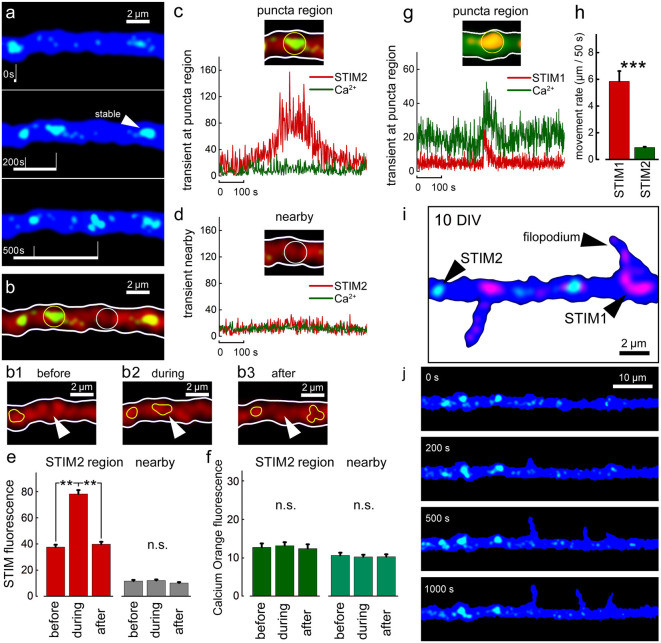
STIM2-YFP puncta movement in relation to [Ca^2+^]_i_ transients. **(A)** Sample illustration of stable and mobile STIM2-YFP (green) puncta over 500 s, DIV 10. Cell morphology marked with EBFP (blue). Overall number of motile/stable puncta per segments of 50 μm: DIV 10: 0.4 ± 0.2/3.3 ± 0.4 (nine cells, *n* = 14 segments); DIV 15: 0.5 ± 0.4/3.4 ± 0.4 (six cells, *n* = 13 segments); DIV 20: 0.9 ± 0.3/4.3 ± 0.2 (six cells, *n* = 10 segments). **(B)** Same dendritic segment with Calcium Orange (CO) fluorescence (red). Two regions are marked with yellow and white circles: with punctum and nearby regions, respectively. Panels **(B1–3)** show the region marked with arrowhead before, during, and after the entrance of the STIM2 punctum (yellow contour). Stable punctum contour is shown on the left. **(C)** Corresponding graph demonstrating the change in STIM2 (red) and CO (green) fluorescence over time inside the marked region. **(D)** A nearby region without STIM2 and the corresponding traces. **(E)** Averaged mobile STIM2 fluorescence before, during, and after the entrance into the region of interest (left, red columns) and in the nearby region (gray). All for DIV 10. *n* = 6 paired region, five cells. **(F)** CO fluorescence for same times and regions as in **(E)**. **(G)** Sample STIM1 puncta fluorescence over time inside the marked region for comparison vs. STIM2: a calcium transient can be detected (Fluo-2, green trace). **(H)** Averaged rate of motility per 50 s, DIV 15, regardless of size puncta, STIM1: 5.9 μm ± 0.8, *n* = 27 representative motile puncta from nine cells; STIM2: 0.9 μm ± 0.1, *n* = 20 representative motile puncta from eight cells. Note that the motility of STIM2 puncta is much lower that of STIM1, difference highly significant, *t* = 6.39, *p* < 0.001. **(I)** Example of a cell at 10 DIV, co-transfected with STIM1 (red) and STIM2 (green). EBFP for cell morphology (blue). **(J)** Example of filopodia spontaneous outgrowth at DIV 10, over 1,000 s in STIM2 transfected neuron (green). Note the lack of association of the growth with STIM2. **Very significant, 0.01 > *p* > 0.001; ***highly significant, *p* < 0.001; n.s., not significant, *p* ≥ 0.05.

A direct comparison between STIMs relations to ambient [Ca^2+^]_i_ variations at different ages was made for both STIM1 and STIM2 (Figure 7). In all cases, comparisons were made with adjacent regions on the dendrites, not endowed with a STIM2 puncta (Figure 7D). There was no correlation between movements of STIM2 puncta along the dendrite and changes in ambient [Ca^2+^]_i_ (Figure 7C), unlike the case with STIM1 (Figure 7G). In general, STIM2 puncta have much lower motility than STIM1 puncta (Figure 7H), or are generally stable (84% of all STIM2 puncta are stable, *n* = 30; representative puncta from six cells, DIV 20). During the formation of new filopodia at DIV 10, STIM2 puncta were not associated with newly formed protrusions (Figure 7J).

Finally, since in earlier studies we and others have proposed that STIM2 is the store calcium sensor, we now compared the response of STIM1 and STIM2 to a reduction in ambient calcium, which has been shown to cause depletion of store calcium. Under these conditions, we expect to detect the movement of STIM molecules to regions in the cytosol across from membranous Orai1. Strikingly, while STIM1 puncta did not change position across 15 min of calcium deprivation (Figure 3), STIM2 appeared to move significantly into filopodia/spines (Figures 3A,C, right panels). Hence, it is likely that this movement is associated with the triggering of calcium influx following its deprivation.

**Figure 8:**
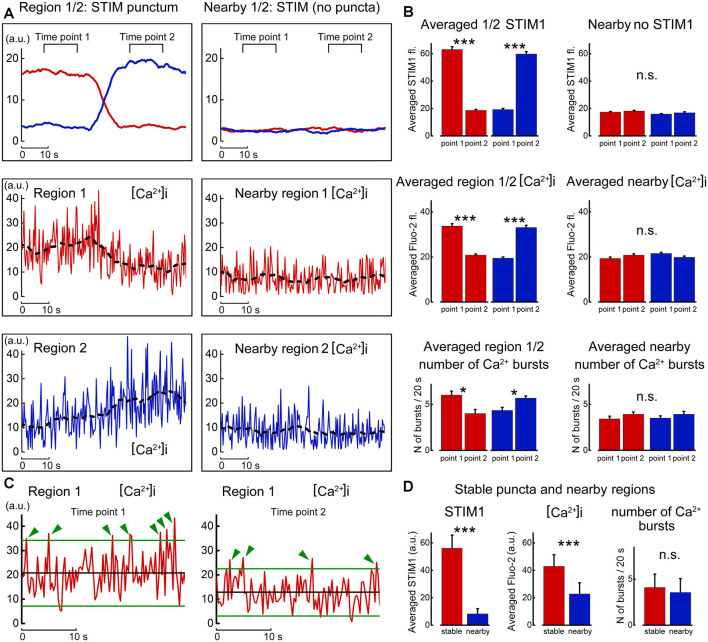
Motility of STIM1 punctum is correlated with local calcium sparks. **(A)** Movement of a STIM1 punctum from region 1 to region 2 (top left, as in Figure 6) and by comparison to an adjacent region, where no STIM1 puncta are detected (top right). Below, calcium fluctuations in the corresponding region 1 and nearby quiet region, and calcium fluctuation in region 2 to where the STIM1 punctum moved. **(B)** Averaged fluorescence of STIM1 and corresponding Fluo2 fluorescence in the two positions where STIM1 was detected. **(C)** Expanded traces taken from region#1 in the presence (left) and absence (right) of STIM1 punctum. Two lines are drawn at 2 SD from the mean, and deviations of the calcium fluorescence line above the regions are marked with green arrows. **(D)** Average fluorescence of STIM1 and Ca^2+^ level in regions of stable puncta and nearby region in 10 s; the number of Ca^2+^ bursts in 20 s, *n* = 9 cells, DIV 15. *Significant, 0.05 > *p* > 0.01; ***highly significant, *p* < 0.001; n.s., not significant, *p* ≥ 0.05.

Finally, the nature of the changes in calcium concentration in relation to STIM1 movement was studied by careful localization of the fluorescent puncta with local calcium variations in the same and adjacent regions of the dendrite (Figure 8). Strikingly, the entry of STIM to the region of interest was accompanied by an increase in spontaneous calcium bursts, akin to those described before as calcium sparks (Ross, 2015). These events were larger than random noise and were seen only in presence of STIM puncta in the region of interest. While the present results do not indicate the mechanism by which STIM1 causes a calcium spark, previous studies suggested such mechanisms (Ross, 2015; Dittmer et al., 2017), and further experiments are required to clarify this possibility in our cultured neurons.

These studies indicate that STIM1, but not STIM2, is associated with local dynamics of [Ca^2+^]_i_ and the formation of novel filopodia. Since filopodia are found predominantly in young neurons, whereas they are converted or replaced by stable spines in older neurons, we compared STIM1- associated filopodia and mature dendritic spines in 1- and 2-week-old cultures (Figure 3). As predicted, STIM1 was associated with filopodia but less with spines in the young cultures, but this distinction was not seen in the older neurons, where STIM2 plays a role in calcium influx, primarily in calcium- starved neurons. This indicates that STIM1 may have a pivotal role in the formation of filopodia in the young cells. This is a logical extension of the assumption that STIM1 may be important especially when there are sparse synaptic connections and little ambient fluctuations of [Ca^2+^]_i_ that is produced in mature spines by afferent activity. In contrast, STIM2 is assumed to underlie the mechanism that is responsible for the influx of calcium when the stored calcium is low, especially in the mature neurons.

## Discussion

STIM1&2 have been shown to play an important role in the regulation of SOCE channels in neuronal and non-neuronal cells. In peripheral (e.g., muscle) cells that are not endowed with a rich diversity of voltage and ligand-gated calcium channels, the ability to regulate [Ca^2+^]_i_, and in parallel, [Ca^2+^] in ER stores is dependent on SOCE channels and the role of voltage-insensitive calcium channel Orai1 is rather critical. The regulation of voltage/ligand-insensitive channels is pretty complicated and demands the presence of a sensor for the estimation of the possible depletion of the calcium stores, and a message to activate the Orai1 channels. This is subserved by the STIM proteins, which in turn, are regulated by septins (Xie et al., 2007; Palty et al., 2012). In neurons, there are two main STIM proteins, STIM1&2, and it was debated which is the dominant form of STIM and what cellular function is it associated with. Our current results suggest that STIM1 is prevalent in young cells where it is associated with calcium influx and formation of filopodia, whereas STIM2 is associated with the more mature neuron, and is associated with SOCE, following calcium starvation.

Our previous results associated STIM2 with Orai1 in the *mature* cultured hippocampal neurons (Korkotian et al., 2017). We were also able to link Orai1 with synaptic plasticity (Tshuva et al., 2017). Assuming that our current results also involve the functioning of Orai1 channels, these results indicate that the STIM1/2-Orai1 coupling is likely to be involved in the early stages of neuronal development and the formation of functional networks.

STIM1 and STIM2 are very similar single transmembrane proteins, and both are calcium-sensors. In the current studies, we indicate that they do not share the same morphological location within the neurons and possibly their functions. This assertion is confirmed by comparing the immunolocalization of the two proteins (e.g., Figure 1), as well as their transfected species. The overlap between the transfected STIMs and their native species indicates that the respective transfected STIMs assume the same functions as the native ones, justifying their use for the analysis of the native STIMs.

We (Fishbein and Segal, 2007) and others (Verbich et al., 2016) have shown that synaptic activity is crucial for the maintenance and stability of neuronal morphology, such that blockade of activity causes the disappearance of dendritic spines and formation and elongation of filopodia. In the present study, we explored this further to find that blockade of activity using TTX can cause STIM-associated increase in spontaneous calcium transients and elongation of nascent filopodia. These observations highlight the role of synapse-independent transient calcium events triggered by STIM1 that are implicit in the functional maturation of dendritic filopodia, akin to what has been described in growth cones (Mitchell et al., 2012; Pavez et al., 2019).

The current results propose that STIM1 is more effective in young cells, that it is more effective in non-active neurons, and that it is associated with filopodia, the immature organelle, more than with dendritic spines, the more stable appendages of the dendrite of a pyramidal neuron. STIM1 puncta are more mobile, and when they move along dendrites, they are associated with the formation of local calcium transients. This is in sharp contrast with STIM2, which appear later in life, are less punctate, and are much less mobile. Thus, the two STIM species are likely to be associated with different functions in the developing and mature neurons, although both are linked to the Orai channel. Thus, two molecules of the same family can be distributed in different cellular compartments, and assume similar roles at different ages of the parent neuron. A hint to such disparity in age/function is in our recent publication (Korkotian et al., 2017) on STIM1/2/Orai localization; when cytosolic calcium is reduced in adult cultured neurons, STIM2 has a much higher tendency to become associated with Orai channels than STIM1. Taken together with our current results, these observations indicate that STIM2 may have a dynamic role, as suggested before, to sense a reduction in store-calcium and move to the plasma membrane to activate Orai to allow an influx of calcium into the store. In contrast, STIM1 may be less tuned to changes in store-calcium but will allow calcium influx in relation to the formation of novel filopodia, as suggested for growth cones (Pavez et al., 2019). The contribution of each molecule species to the ongoing [Ca^2+^]_i_ regulation needs further exploration.

The comparison between [Ca^2+^]_i_ evoked by synaptic activity and by apparent activation of the STIM1/Orai coupling is interesting indeed. The synaptically-evoked rise in [Ca^2+^]_i_ is rather large, but rise time and decay are much faster than STIM1 associated [Ca^2+^]_i_ rise. Both types of changes are estimated by the same high-affinity calcium sensor, Fluo-2AM, and so the difference in kinetics can be attributed to a different affinity of the sensor. The slower kinetics of the STIM1-evoked [Ca^2+^]_i_ reflects the lower magnitude of change, and the need to accumulate a significant amount of calcium to allow activation of calcium-dependent kinases, which will lead eventually to synaptic growth and plasticity.

The functional importance of the STIM/Orai complex has been studied in relation to its possible involvement in neurodegenerative Alzheimer’s disease (AD). Both STIM2 (Sun et al., 2014; Zhang et al., 2015) and STIM1 (Zeiger et al., 2013) have been implicated in the regulation of amyloid-beta peptide, which has been shown to accumulate in AD (Zeiger et al., 2013), and have been shown to regulate the formation of dendritic spines in a mouse model of AD (Sun et al., 2014; Zhang et al., 2015). Since our results indicate that the two STIM species can reside side by side, at different ages and cellular locations, possibly a deficient STIM1 peptide may alter properties of SOCE channels, and consequently their involvement in the regulation of calcium stores in AD. However, our studies are conducted in very young neurons, compared to the human case and the mouse model, and so the proposal that STIMs are involved in the generation of AD needs further experimentation to explore this link further and to propose channels for the restoration of calcium deficiency in this disease.

## Data Availability Statement

The raw data supporting the conclusions of this article will be made available by the authors, without undue reservation.

## Ethics Statement

The animal study was reviewed and approved and this study was carried out in accordance with the recommendations of the IACUC committee of the Weizmann Institute, which is adhered to the Israeli law for handling of research animals. The protocol was approved by the IACUC committee of the Weizmann Institute.

## Author Contributions

EK and MS designed the experiments. EK and LK conducted the experiments and analyzed the data. EK, LK, and MS wrote the manuscript. All authors contributed to the article and approved the submitted version.

## Conflict of Interest

The authors declare that the research was conducted in the absence of any commercial or financial relationships that could be construed as a potential conflict of interest.
